# Cuproptosis key gene FDX1 is a prognostic biomarker and associated with immune infiltration in glioma

**DOI:** 10.3389/fmed.2022.939776

**Published:** 2022-11-29

**Authors:** Hanwen Lu, Liwei Zhou, Bingchang Zhang, Yuanyuan Xie, Huiyin Yang, Zhanxiang Wang

**Affiliations:** ^1^Department of Neurosurgery, Xiamen Key Laboratory of Brain Center, The First Affiliated Hospital of Xiamen University, School of Medicine, Xiamen University, Xiamen, China; ^2^Department of Neuroscience, Institute of Neurosurgery, School of Medicine, Xiamen University, Xiamen, China; ^3^Department of Neurosurgery, The First Affiliated Hospital of Xiamen University, School of Medicine, Xiamen University, Xiamen, China

**Keywords:** Cuproptosis, glioma, prognostic biomarker, immune infiltration, lipoylation

## Abstract

Recent studies have found that the protein encoded by the *FDX1* gene is involved in mediating Cuproptosis as a regulator of protein lipoylation and related to immune response process of tumors. However, the specific biological function of *FDX1* in glioma is currently unclear. To explore the potential function of *FDX1*, this study explored the correlation between the expression of *FDX1* in cancers and survival prognosis by analyzing the public databases of GEPIA and Cbioportal. Immune infiltration was analyzed by the TIMER2.0 database in tumors. The possible biological processes and functions of FDX1-related in glioma were annotated through gene enrichment. Relationship between Cuproptosis and autophagy was explored through gene co-expression studies. Summary and conclusions of this study: (1) FDX1 is highly expressed in gliomas and associated with poor prognosis in low-grade gliomas (LGG). (2) Gene annotation indicates that FDX1 is mainly involved in the tumor protein lipoylation and cell death. (3) *FDX1* expression is positively correlated with the infiltration of immune cells. (4) *LIPT2* and *NNAT*, two other genes involved in lipoylation, may be unidentified marker gene for Cuproptosis. And the Cuproptosis genes related to FDX1 were positively correlated with the expression of autophagy marker genes *Atg5*, *Atg12*, and *BECN-1*. This evidence suggests that there may be some interaction between FDX1 mediated Cuproptosis and autophagy. In summary, FDX1 may serve as a potential immunotherapy target and prognostic marker for Glioma.

## Introduction

Glioma is one of the most common central nervous system malignancies (1–6). Evidence shows that high-grade glioma (HGG) has obvious aggressiveness and heterogeneity (7–14). In 2022, the World Health Organization (WHO) updated the classification criteria of glioma according to the molecular characteristics of different gliomas, which will help patients obtain more accurate diagnoses and precise personalized treatment in clinical practice (1, 2). Therefore, exploring new molecular marker is important work. FDX1 is a mitochondrial-associated protein, named ferredoxin 1 (FDX1) for it is closely related to iron-sulfur protein synthesis (15–17). Meanwhile, it acts as a key regulator of lipoylation to regulate protein lipoylation process (18, 19). It is worth noting that a recent report found that *FDX1*, as a key enzyme, regulates the Cuproptosis by regulating protein lipoylation process (20).

However, the role and biological function of *FDX1* in gliomas are currently unclear. Currently, it is generally accepted that the tumor microenvironment (TME) is one of the key factors in tumor initiation and progression (7, 21–29). It is mainly composed of tumor cells, fibroblast cells, immune cells, various signal molecules, extracellular matrix, special physical and chemical factors (30–33). Evidence shows that the tumor microenvironment significantly affects tumor diagnosis, survival outcomes, and clinical treatment sensitivity (21, 23, 33–35). In recent years, related immunological studies have found that immune cell infiltration plays a key role in the tumor microenvironment in the formation, occurrence and development (31, 36–47). The most concerned are the immune checkpoint protein PD-1 and its ligands PD-L1 and PDCD1LG2, tumor transforming growth factor B1 (TGFB1) and its receptors TGFB1R (48–54). TGFB1 plays an immunosuppressive role in the process of tumor progression. Inhibiting the activation and differentiation of B lymphocytes and T lymphocytes, further leads to the immune dysfunction of the body, allowing tumor cells to escape the surveillance of the immune system (55–57).

This study aimed to explore the relationship and possible signaling pathways between *FDX1* expression and the prognosis of glioma patients utilizing bioinformatics. In addition, by analyzing the expression correlation between immune cell signatures and *FDX1* expression, we explored the relationship between *FDX1* expression and the infiltration of immune cells in the tumor microenvironment and further clarified whether FDX1 could be used as a new type of glioma patient immunotherapy markers. At the same time, potential Cuproptosis mediators and whether Cuproptosis and ferroptosis have common features in autophagy dependence were identified by gene co-expression research method.

## Materials and methods

### Public database

The patient transcriptome data and corresponding clinical information used in this study were derived from the Chinese Glioma Patient Genome Atlas (CGGA,^1^) (58) and The Cancer Genome Atlas (TCGA, see text footnote 1) public database. Gene annotation and differential gene analysis are completed by the GENEMAINA^2^ data platform (GENEMAINA is a visualization platform that integrates a large amount of annotation information and gene interactions, which can identify co-expressed genes of specific gene in tumors. Gene functions and signaling pathway informations can be predicted through the annotated informations (59).

The mutation information analysis of the *FDX1* gene was completed by the cBioPortal^3^ platform TCGA-GBM and TCGA-LGG datasets. Correlation between the *FDX1* gene and the level of tumor immune infiltration was performed by the analysis tool TIMER2.0. TIMER2.0 is an immune assessment tool constructed based on tumor gene signatures expression in TCGA, which can be used to assess the correlation between different genes and immune cell subtypes, as well as the immune level and purity of infiltration.^4^ LinkedOmics is an analysis tool for identifying differentially expressed genes associated with FDX1. In this study, the statistical methods used were all tested and distinguished by Pearson correlation coefficient (60). The relationship between the *FDX1* gene and immune-related factors, as well as the GO annotation of the gene and the KEGG signaling pathway enrichment analysis, were completed by the TISIDB database. TISIDB is an analysis platform to study the interaction between genes and tumor immunity^5^ (61).

### Gene set enrichment analysis

In this study, the online tool LinkedOmics was used to analyze potential genes related to *FDX1* function. According to the expression abundance of key genes, which were divided into high and low expression groups. GSEA^6^ module was employed to cellular process enrichment, and GSEA enrichment was estimated using the normalized enrichment score (FDR ≤ 0.25, *p* ≤ 0.05 indicating a statistical difference).

### Statistical analysis

The statistical methods used in this study were Spearman’s tests to evaluate the significance and correlation between the expressed genes. 50% of the gene expression value was set as the critical point, and all sample were divided into two groups according to the expression level. The differences and significance of survival rates among the groups were further evaluated by the Kaplan-Meier algorithm. ANOVA analysis was used to count the expression of *FDX1* gene in three independent datasets in the CGGA database.

## Results

### Pan-cancer expression analysis of *FDX1* gene

**FIGURE 1:**
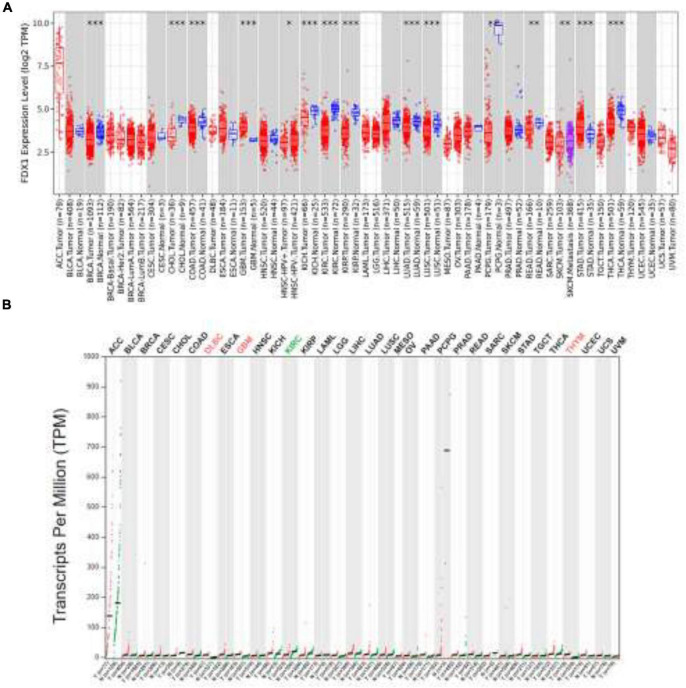
Expression of *FDX1* gene in different cancers. **(A)** Expression of *FDX1* gene in different cancer and normal tissues of TIMER2.0 database (*P ≤ 0.05, **P ≤ 0.01, ***P ≤ 0.001). **(B)** Expression of FDX1 Transcripts in different cancer and normal tissues in GEPIA database (*P ≤ 0.05, **P ≤ 0.01, ***P ≤ 0.001).

To understand the expression of the *FDX1* gene in cancers, TIMER2.0 was used to analyze the transcriptome expression level of the *FDX1* gene in different tumors and normal tissues. The results showed that the *FDX1* gene was abnormally expressed in most tumor tissues compared to normal tissues. It is worth noting that the expression of the FDX1 gene was abnormally high in glioma than normal tissues, especially in glioblastoma (GBM), the expression level was the highest (Figure 1A). Next, the expression level of FDX1 in different tumor and normal tissues was further evaluated through the GEPIA online database (Figure 1B). The results showed that the transcripts level of FDX1 in GBM and Low grade glioma (LGG) was significantly higher than normal tissues. This is consistent with the conclusions obtained from TIMER2.0.

### Abnormally expression of *FDX1* gene in gliomas

**FIGURE 2:**
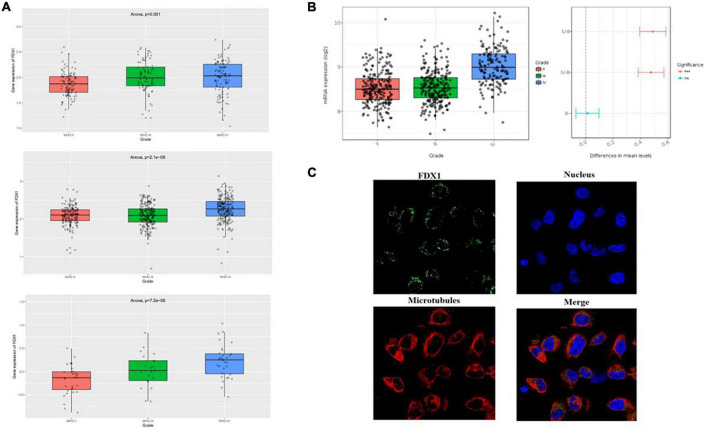
Expression level analysis of *Fdx1* gene in multiple public databases. **(A)** Expression of *Fdx1* gene in three independent research cohorts of CGGA. **(B)** Expression of *Fdx1* gene in TCGA dataset (LGG-GBM). **(C)** Expression of FDX1 in glioma cell line U251 cells (the data from Human Protein Altas public database).

The present study analyzed three independent transcriptome datasets in the CGGA database, and the results showed that *FDX1* gene expression was higher in HGG relative to LGG (Figure 2A). To further check the accuracy of the above results, expression levels of FDX1 were analyzed using the glioma transcriptome datasets published by different experimental groups provided by the Gliovis platform.^7^ The results showed that FDX1 was abnormally up-regulated in both TCGA and Rembrandit datasets relative to LGG and HGG samples (Figure 2B). The results are consistent with previous. Meanwhile, we analyzed the expression of FDX1 protein in glioma cells by HPA (Human Protein Database)^8^. The results showed that FDX1 protein was expressed in the glioma cell line U251 cells, and was mainly expressed in the cytoplasm and cell membrane (Figure 2C).

**FIGURE 3:**
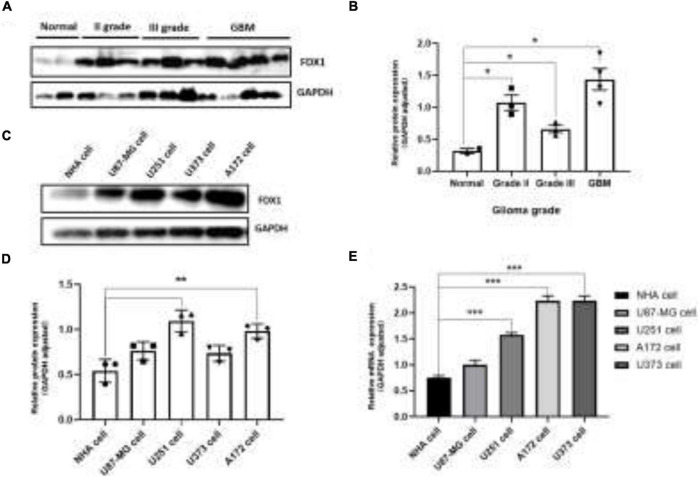
Expression level analysis of FDX1 in glioma tissue glioma cell lines. **(A)** Expression of FDX1 protein in Normal and glioma tumor tissue (Grade II, Grade III, and GBM). **(B)** Statistical analysis of FDX1 protein relative to GAPDH gene expression in normal tissues and glioma tissues. **(C)** Expression of FDX1 protein in normal glial cell line (NHA cell) and glioma cell lines (U87-MG cell, U251 cell, U373 cell, and A172 cell). **(D)** Statistical analysis of FDX1 relative to GAPDH gene expression J in glia cell line and glioma cell lines. **(E)** mRNA expression of FDX1 gene in normal glial cell (NHA cell) and glioma cell lines. (*P ≤ 0.05, **P ≤ 0.01, and ***P ≤ 0.001).

To further verify our analysis results, we detected the expression of FDX1 in glioma tissues and cell lines. The results showed that the protein expression of FDX1 in grade II, III, and GBM of glioma was significantly up-regulated compared with the normal group (Figures 3A,B). The results showed that the protein expression of FDX1 in Normal glial cell (NHA cell) and glioma cell lines (U87-MG, U251, U373, and A172) was significantly up-regulated compared with the normal group (Figures 3C,D). In order to further prove our conclusion, we also detected the mRNA expression of *FDX1* gene in NHA cell and glioma cell lines. The results showed that the mRNA expression of FDX1 in U251, U373, and A172 cell were significantly higher than NHA cell (Figure 3E).

To further understand the relationship between the expression of *FDX1* gene and the clinical characteristics of glioma patients, we performed univariate and multivariate regression analysis. The results showed that in CGGA database, *FDX1* expression was statistically correlated with patient age, tumor grade, chemotherapy resistance, IDH mutation and 1p19q codeletion. The results in TCGA database showed that the expression of FDX1 was statistically correlated with tumor grade and 1p19q codeletion (Tables 1, 2).

**TABLE 1:** Cox regression analysis of the clinical variables, and overall survival in CGGA cohorts.

Variables	Univariates		Multivariates	
	HR (95% CI for HR)	*P*-value	HR (95% CI for HR)	*P*-value
FDX1	1.720 (1.453–2.037)	<0.001*	1.299 (1.104–1.528)	<0.002*
Age	1.622 (1.343–1.958)	<0.001*	1.267 (1.037–1.547)	<0.021
Gender	1.046 (0.867–1.261)	0.639	1.079 (0.892–1.307)	0.433
Grade	2.884 (2.527–3.292)	<0.001*	2.790 (2.041–3.814)	<0.001*
Radio	0.928 (0.719–1.197)	0.565	0.842 (0.641–1.105)	0.216
Chemo	1.645 (1.326–2.041)	<0.001*	0.659 (0.517–0.840)	<0.001*
IDH-mutation	0.317 (0.262–0.384)	<0.001*	0.621 (0.492–0.783)	<0.001*
1p19q_codeletion	0.231 (0.169–0.315)	<0.001*	0.413 (0.296–10.578)	<0.001*

**TABLE 2:** Cox regression analysis of the clinical variables, and overall survival in TCGA cohorts.

Variables	Univariates		Multivariates	
	HR (95% CI for HR)	*P*-value	HR (95% CI for HR)	*P*-value
FDX1	1.088 (0.815–1.452)	0.567	1.028 (0.765–1.381)	0.855
Age	2.039 (1.397–2.977)	0.5	1.608 (1.075–2.408)	0.021
Gender	0.774 (0.611–0.982)	0.035	0.765 (0.596–0.980)	0.034
Grade	1.512 (1.329–1.722)	<0.001*	1.386 (1.208–1.590)	<0.001*
Radio	0.685 (0.516–0.908)	0.008	0.804 (0.579–1.115)	0.19
Chemo	0.884 (0.698–1.120)	0.308	0.952 (0.728–1.245)	0.72
IDH-mutation	0.699 (0.309–1.579)	0.389	1.616 (0.682–3.828)	0.275
1p19q_codeletion	0.019 (0.003–0.145)	<0.001*	0.018 (0.002–0.151)	<0.001*

### The prognostic value of FDX1 in glioma patients

**FIGURE 4:**
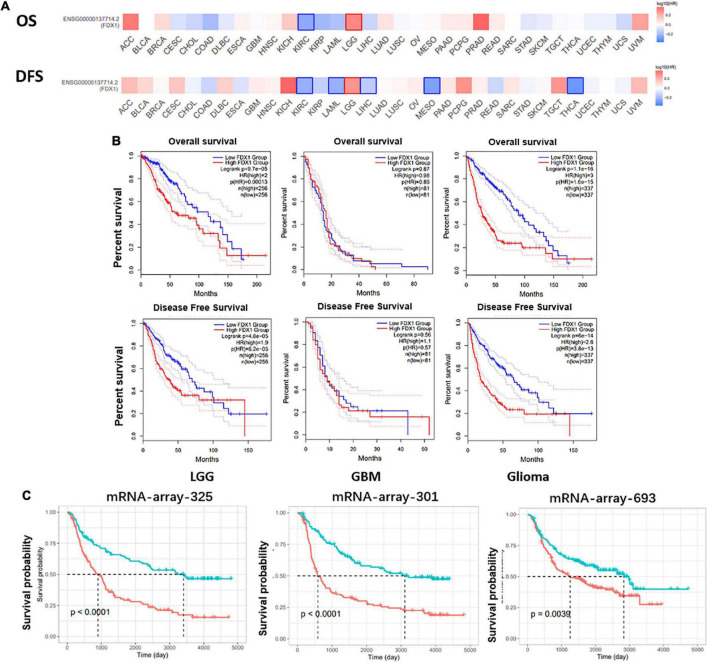
Potential prognostic value of FDX1 in glioma. **(A)** The overall survival and progression-free survival curve of FDX1 in glioma. **(B)** Survival analysis of glioma in TCGA database. **(C)** Survival analysis of glioma in CGGA datasets.

To further explore the correlation between FDX1 expression and clinical characteristics of glioma patients. Online tool GEPIA^9^ was used to performed the survival analysis of LGG and GBM samples. The results showed that FDX1 expression was associated with overall survival (OS) and progression-free survival (PFS) in patients with low-grade glioma, but not with OS or PFS in GBM patients (Figure 4A). Further, we separately analyzed the correlation between FDX1 expression and prognosis in all glioma patients, LGG and GBM patients. The results showed that high expression of FDX1 was significantly associated with the prognosis time of all glioma patients. FDX1 expression is significantly associated with prognosis in LGG. However, the prognosis is not significantly different from that of GBM (Figure 4B). At the same time, we verified the result in three independent cohorts of CGGA, and the conclusion indicate that high expression of FDX1 gene was associated with the overall prognosis of glioma (Figure 4C).

### Analysis of the expression of FDX1 in glioma

**FIGURE 5:**
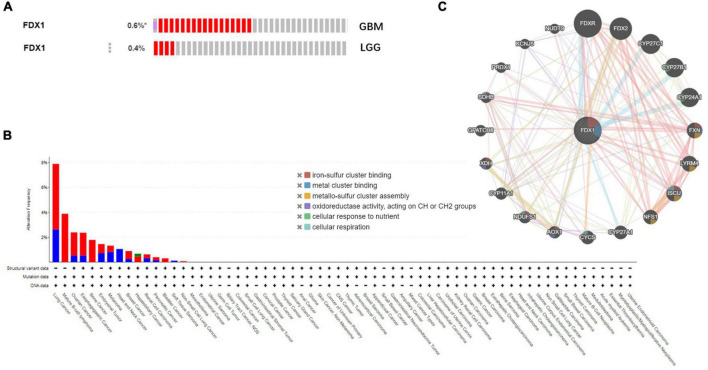
Genetic alterations and interactions of *FDX1* gene in glioma. **(A)** Genetic alterations of *FDX1* gene in cBioPortal database. **(B)** Mutation frequency of *FDX1* gene in GBM and LGG samples. **(C)** Interaction network analysis of *FDX1* gene was performed by GeneMANIA.

To further understand the relationship between *FDX1* gene mutations and tumor clinical characteristics, we used the cBioPortal platform to analyze the frequency mutations in 33 cancers from the TCGA database. The results showed that *FDX1* had a lower mutation frequency in tumors (Figure 5A). The mutation frequencies were 0.6 and 0.4% in GBM and LGG samples (Figure 5B). At the same time, *FDX1* gene interaction network constructed by GeneMANIA database. The results suggested that FDX1 may be closely related to redox homeostasis, nutritional stress and assembly process of oxygen-sulfur cluster complexes (Figure 5C).

### *FDX1* gene co-expression network construction in glioma

**FIGURE 6:**
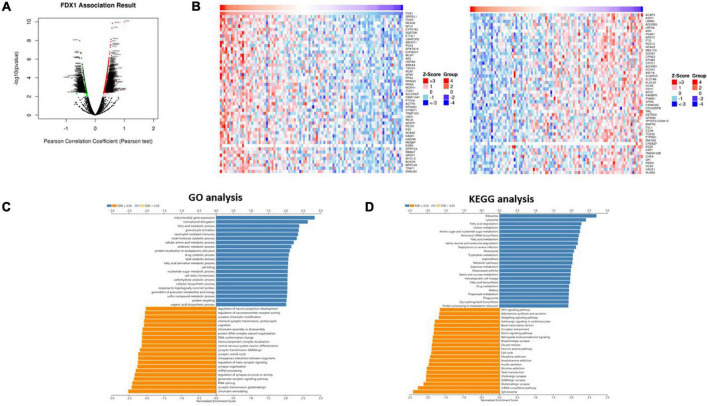
Co-expression network of FDX1 gene in glioma. **(A)** The scheme showed FDX1-related differentially expressed genes in glioma. **(B)** The scheme showed FDX1-related genes in glioma. **(C)** Genes of FDX1 co-expressed enrichment analysis performed by LinkedOmics (GSEA method).

To further explore the function of *FDX1* in glioma, we used the LinkedOmics database to analyze the gene co-expression network centered on *FDX1* in glioma. As shown below, in the volcano plot, red represents positive correlated genes co-expressed with FDX1, and the green represents negatively correlated genes co-expressed with *FDX1* (Figure 6A). We selected genes in the top 50% for further correlation analysis and visualized them with a heatmap (Figure 6B). Gene GO annotation and KEGG pathway enrichment analysis (GSEA) results showed that *FDX1* and its co-expressed genes were mainly related to cellular protein lipoylation, lipid metabolism, small molecule metabolism and immune response (Figures 6C,D).

### Correlation between *FDX1* gene expression and immune infiltration level in Glioma

**FIGURE 7:**
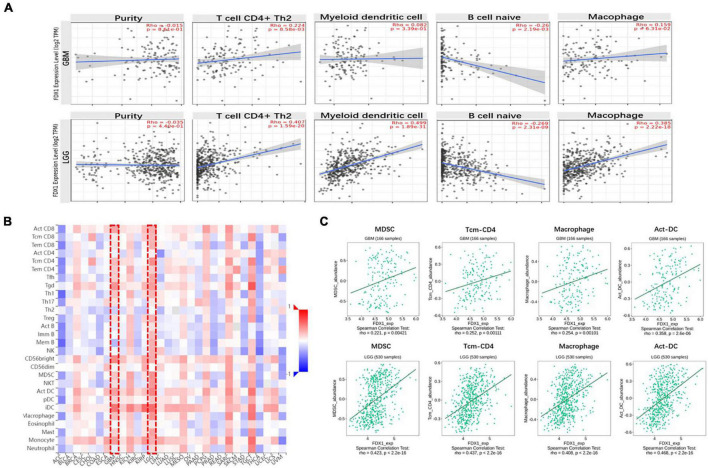
The relationship between *FDX1* gene expression and immune infiltration level in glioma. **(A)** Expression of *FDX1* gene related to immune infiltration of CD4 + T cells, Dendritic cells, B cells and macrophage cells in glioma. **(B)** Analysis between *FDX1* gene expression and immune infiltration levels by TISIDB. **(C)** Correlations between *FDX1* gene expression and Dendritic cells, effector memory CD4 T cells, Myeloid-derived suppressor cells and macrophage cells infiltration in glioma.

Next, to clarify the relationship between *FDX1* gene expression and immune cell infiltration in the tumor microenvironment, the TIMER2.0 platform were used to analyze different dimensions of immunity. The results showed a positive correlation between *FDX1* expression and infiltration of CD4 + positive T cells, T lymphocytes, Macophage cells and dendritic cells, and negative correlation with B cells (Figure 7A). We further analyzed the relationship between *FDX1* expression and immune cell infiltration through the TISIDB database. The results showed that *FDX1* expression was positively correlated with myeloid-derived suppressor cells, CD4 + T central memory T cells, Macrophage cells, and active Dendritic cells (Figures 7B,C).

**FIGURE 8:**
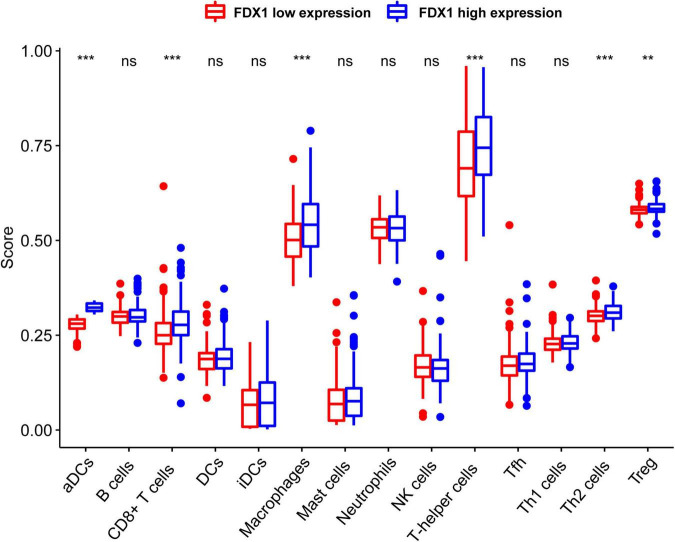
The expression abundance of immune infiltrating cells in Fdx1 high and low expression group was calculated by ssGSEA method (single sample GSEA). *P* ≤ 0.05, **P ≤ 0.01, and ***P ≤ 0.001.

In order to know more about the relationship between FDX1 expression and immune cells, we analyzed the expression difference of immune cells between the high expression group and the low expression group of FDX1 gene through ssGSEA. The results showed that in FDX1 overexpression group, aDCs (dendritic cells), CD8+ T cells, helper T cells, Th1 cells (helper type 1 T cells) and Treg cells were significantly up-regulated (Figure 8).

### The relationship between *FDX1* gene and immune microenvironment markers expression in glioma

**FIGURE 9:**
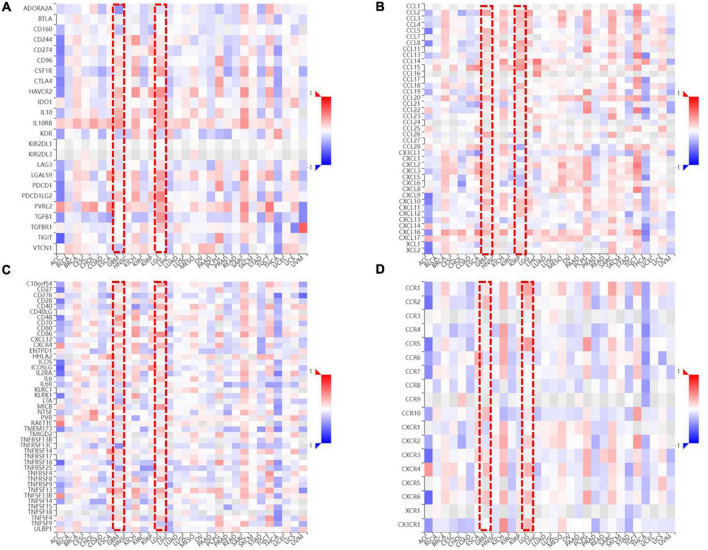
The relationship between *FDX1* and immune-related genes expression. **(A)** The relationship between *FDX1* and immunosuppressive genes expression in glioma. **(B)** The relationship between *FDX1* and glioma immunostimulatory genes expression in glioma. **(C)** The relationship between *FDX1* and chemokine genes expression in gliomas. **(D)** The relationship between *FDX1* and receptor genes expression in glioma.

To clarify the relationship between FDX1 expression and immune microenvironment. Correlation between FDX1 gene expression and immune regulatory genes, and chemokines was analyzed using the TISIDB online tool. The results suggest there was a correlation between *FDX1* and immunosuppressive gene expression. The five inhibitor genes that were highly correlated with FDX1 included HAVCR2, TGFB1, CD96, IL10, and IL10Rb (Figure 9A). The genes CD86, CXCR4, MICB, and TNFRSF9 were also positively correlated with FDX1 (Figure 9B). In addition, we analyzed correlation between FDX1 expression with chemokines and apoptosis. The top four significantly positively correlated chemokines included CCL2, CCL8, CXCL14, and CXCL15 (Figure 9C). We also analyzed the relationship between FDX1 expression and chemokine receptors, and the top four receptors included CCR1, CCR5, CXCR4, and CX3CR1 (Figure 9D). Based on the above information, we speculate *FDX1* may be an important immune regulatory gene.

### *FDX1* co-expression identifies potential Cuproptosis genes and relationship to autophagy

Since the key genes and important regulatory mechanisms of Cuproptosis are still in the preliminary stage of exploration, the upstream regulators of FDX1, downstream effector proteins, Cuproptosis signals transduction and the specific lethal molecular mechanisms have not yet been elucidated. Available evidence indicate that Cuproptosis of tumor cells mainly depend on protein lipoylation process. However, only 10 key genes have been identified so far, and the identification of more Cuproptosis key genes is of great significance for further elucidating its molecular mechanism. It is also due to the programmed death “ferroptosis” induced by metal ions homeostasis inblanced, which is generally considered to be an autophagy-dependent programmed death process. This phenomenon enlightens us, on whether the Cuproptosis process is also an autophagy-dependent death process?

Although, recent reports and our enrichment results in this study indicate that FDX1 expression is associated with cell death. But there are still many questions that remain unexplained. To further explore the potential key genes of Cuproptosis and whether there is a correlation between Cuproptosis and autophagy, we analyzed the relationship between FDX1-related genes through gene co-expression network analysis methods. Correlations between lipoylation and autophagy-related genes.

**FIGURE 10:**
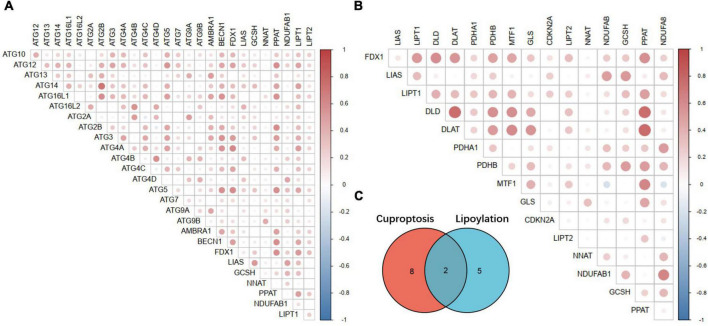
Correlation analysis between key Cuproptosis genes, autophagy-related genes and lipoylation genes. **(A)** Correlation analysis between Fdx1-related Cuproptosis genes and autophagy-related genes. **(B)** Correlation analysis between Fdx1-related Cuproptosis genes and key genes of protein lipoylation. **(C)** Illustration of the intersection of identified Cuproptosis and protein lipoylation genes.

Interestingly, except identified lipoylation process genes *LIAS, LIPT1, DLD, DLAT, PDHA1, PDHB, MTF1, GLS*, and *CDKN2A*. Two other key genes of lipoylation, *LIPT2* and *PPAT*, may be potential Cuproptosis regulator genes, which may be downstream effectors of *FDX1* according to the annotation results (Figures 10B,C). At the same time, by analyzing the correlation between the *FDX1* gene and autophagy genes, we found that *FDX1* was significantly positively correlated with the expression of key genes *Atg5*, *Atg12*, *BECN-1*, and *Atg16L* in the autophagy process (Figure 10A). This information suggests that Cuproptosis, similar to ferroptosis, may be a programmed death of cells associated with the development of autophagy.

## Discussion

As an vital part of the tumor microenvironment, multiple pieces of evidence have shown that immune cells in the microenvironment are involved in regulating tumor invasion and progression. Due to the recent performance of immunotherapy in clinical applications, immunosuppressants for specific targets represented by PD1 and PD-L1 have been successfully developed. For example, monoclonal antibodies or bispecific antibodies corresponding to antigens such as EGFR, VEGFR, PD-L1, TGFB1, and CTLA-4 have been used to treat tumors, such as acute lymphoma leukemia, colorectal cancer, and breast cancer (62–68). Although according to the existing reports and actual clinical manifestations, the benefit of immunotherapy for patients with brain tumors, especially high-grade gliomas, is limited (66, 69–73). Therefore, we speculate that the heterogeneity of the tumor microenvironment is one of the important factors limiting glioma patients’ benefit from biologically targeted therapies (70–72).

Through the analysis of data from CGGA and TCGA, we found a positive correlation between FDX1 expression and glioma grade. Further, we performed gene annotation on the co-expressed genes of *FDX1* in glioma, GO annotation, and KEGG signaling pathway enrichment analysis showed that FDX1 expression is closely related to immune response and inflammation. In this study, we also found that high FDX1 expression was associated with the infiltration of immune cells, including MDSC, Tcm-CD4 cells, macrophage cells and Act-DCs in GBM and LGG.

According to current reports and consensus, the effectiveness of tumor immunotherapy mainly depends on the immune microenvironment of the tumor. One of the key factors affecting the immune microenvironment is the expression of immune checkpoint genes. By exploring the relationship between the *FDX1* gene and immune checkpoint genes, we found that there is a strong co-expression relationship between the *FDX1* gene and immune checkpoint Suppressor gene TGFB1 in glioma. This evidence suggests that *FDX1* expression may be associated with the up-regulation of immune checkpoints.

Previous reports have found that abnormal aggregation of lipoylated proteins interferes with iron-sulfur cluster proteins in the respiratory chain complex, resulting in proteotoxic stress and cell death. FDX1 is an important regulator in the process of protein lipoylation, and its abnormal function may be related to some cell death. It is noteworthy that, Zhang Z et al. found that *FDX1* gene was associated with the prognosis of Lung adenocarcinoma (LUAD) and FDX1 can promote ATP production (74). Zhang C et al. found that the expression of FDX1 has prognostic value for the survival of Adrenocortical Cancer (ACC), Kidney Clear Cell Carcinoma (KIRC), Head and Neck Cancer (HNSC), Thyroid Cancer (THCA), and LGG. In addition, the expression level of FDX1 was confirmed to be closely related to immune infiltration (75). Zhang Y. et al. found that *FDX1* is an independent prognostic factor and potential prognostic biomarker of WHO grade II/III glioma (76). Wang X et al. found that the high expression of *FDX1* was significantly correlated with the overall survival rate of Renal Cell Carcinoma (RCC) (*p* < 0.05). Variable regression analysis showed that the high expression of *FDX1* was an important independent predictor of overall survival, which could be used as a potential prognostic indicator and therapeutic target for RCC (77). Zhang et al. found that the overall survival rate and disease-specific survival rate of colon adenocarcinoma patients (COAD) in the FDX1 high-expression group were better than low expression group. GO-KEGG enrichment analysis showed that FDX1 and its co-expressed genes were related to the pathogenesis of COAD. In addition, the expression of FDX1 in COAD was positively correlated with “inflammation level”. The expression of FDX1 was positively correlated with the infiltration level of CD8^+^T cells, NK cells and neutrophil cells but negatively correlated with CD4^+^T cells and cancer associated fibroblasts (78).

Recent blockbuster reports have found that FDX1, a key regulator of Cuproposis, regulates cell death by influencing fatty protein lipoylation. To further explore the role of FDX1 in gliomas, we attempted to identify potential Cuproptosis key genes by bioinformatics analysis, comparing the co-expression gene of FDX1 in gliomas with with an additional 6 lipoacylation-related genes (79–82). The key genes were subjected to intersection analysis and expression correlation analysis, to screen and identify potential Cuproptosis process genes through co-expression network research methods. Our results show that *LIPT2* and *NNAT* and lipoylation genes such as *LIAS* and *GLS* have been reported to be strongly correlated with FDX1 expression, but not identified in this report. Therefore, we speculate that *LIPT2* and *NNAT* may be potential key genes for Cuproptosis. Further experimental identification needs to be verified in the follow-up work.

Similar to ferroptosis, Cuproptosis is also a programmed cell death process induced by excessive accumulation of metal ions. More and more reports show that the ferroptosis of cells is an autophagy-dependent programmed death. To further explore the relationship between Cuproptosis and autophagy, we analyzed the correlation between the molecular markers of the Cuproptosis process represented by *FDX1* and the key genes of autophagy in glioma by co-expression network analysis. Consistent with our expectations, the key genes for Cuproptosis and autophagy key genes, such as *Atg5*, *Atg12*, and *BECN-1*, were co-expressed and strongly correlated. This evidence suggests that there may be some correlation between the two. Therefore, we boldly put forward the hypothesis that as metal ion toxicity induces programmed cell death, autophagy is also a pre-stress process of Cuproptosis. When cells fail to regulate cellular homeostasis through autophagy to ensure normal cell operation, they switch to the activation of the copper ionophore receptor protein FDX1, which initiates the toxicity-induced Cuproptosis process.

The innovation of this study is that, for the first time, we found that FDX1 in glioma is associated with poor patient prognosis, and also explored the possible mechanism of FDX1 in glioma involved in the immune microenvironment. We further confirmed the correlation of FDX1 with glioma immune infiltration and proposed that FDX1 may serve as a novel immunotherapy biomarker. Therefore, our results will provide a certain reference for immunotherapy of glioma in the future. It is worth mentioning that we discovered the underlying genes *LIPT2* and *NNAT* for Cuproptososis through co-expression analysis. We speculate that there is a certain correlation between Cuproposis and autophagy, but whether the correlation is as autophagic dependent as ferroptosis is more experimental evidence to prove in the future.

## Data availability statement

The datasets presented in this study can be found in online repositories. The names of the repository/repositories and accession number(s) can be found in the article/Supplementary material.

## Author contributions

HL: conceptualization, formal analysis, and manuscript writing. LZ and HY: data curation. ZW: funding acquisition. BZ and YX: manuscript review. All authors contributed to the article and approved the submitted version.

## Footnotes

1
http://www.cgga.org.cn/

2
http://genemania.org/

3
http://www.cbioportal.org/

4
https://cistrome.shinyapps.io/timer/

5
http://cis.hku.hk/TISIDB/index.php

6
http://software.broadinstitute.org/gsea/index.jsp

7
http://gliovis.bioinfo.cnio.es/

8
https://www.proteinatlas.org/

9
http://gepia.cancer-pku.cn/
