# Corrigendum: Cuproptosis key gene FDX1 is a prognostic biomarker and associated with immune infiltration in glioma

**DOI:** 10.3389/fmed.2023.1244638

**Published:** 2023-07-10

**Authors:** Hanwen Lu, Liwei Zhou, Bingchang Zhang, Yuanyuan Xie, Huiyin Yang, Zhanxiang Wang

**Affiliations:** ^1^Department of Neurosurgery, Xiamen Key Laboratory of Brain Center, The First Affiliated Hospital of Xiamen University, School of Medicine, Xiamen University, Xiamen, China; ^2^Department of Neuroscience, Institute of Neurosurgery, School of Medicine, Xiamen University, Xiamen, China; ^3^Department of Neurosurgery, The First Affiliated Hospital of Xiamen University, School of Medicine, Xiamen University, Xiamen, China

**Keywords:** Cuproptosis, glioma, prognostic biomarker, immune infiltration, lipoylation

In the published article, there was an error in affiliation 1. Instead of “Department of Neurosurgery, Xiamen Key Laboratory of Brain Center, The First Affiliated Hospital of Xiamen University, Xiamen, China”, it should be “Department of Neurosurgery, Xiamen Key Laboratory of Brain Center, The First Affiliated Hospital of Xiamen University, School of Medicine, Xiamen University, Xiamen, China”.

In the published article, there was an error in affiliation 2. Instead of “Institute of Neurosurgery, School of Medicine, Xiamen University, Xiamen, China”, it should be “Department of Neuroscience, Institute of Neurosurgery, School of Medicine, Xiamen University, Xiamen, China”.

In the published article, there was an error in affiliation 3. Instead of “Department of Neurosurgery, The First Affiliated Hospital of Xiamen University, Xiamen, China”, it should be “Department of Neurosurgery, The First Affiliated Hospital of Xiamen University, School of Medicine, Xiamen University, Xiamen, China”.

The authors apologize for these errors and state that this does not change the scientific conclusions of the article in any way. The original article has been updated.

